# A German Clergyman on Quacks

**Published:** 1907-01

**Authors:** 


### SELECTIONS AND ABSTRACTS.

### A GERMAN CLERGYMAN ON QUACKS.

The German popular magazine, *Der Deutsche*, contained in a recent issue an article by a clergyman, Pfarrer E. Grucker, describing the way in which he could follow the trail of certain quacks through many sick rooms in the various parishes he has had in his charge. The people were not always convinced that the quack's prescription would help them, "but at least," they said, "if it don't help it will do no harm." In one instance leg ulcers were treated with a swallow's nest mixed with pigeon manure and oil, applied on a piece of uncleaned wool taken from between the legs of a living sheep. Felons were tretad by tying an agleworm around the finger, "that the worm outside hight kill the worm inside causing the trouble." The diagnosis was made by one popular irregular from an article of clothing brought from the sick person, for whom he then prescribed with confidence. Many of the strange, hocus-pocus performances ordered by the quacks are described as Grucker had occasion to observe their execution. He adds: "But not only in the backwoods villages were the quacks found to be rampant. Even in the great cities there are many dark corners where fortune tellers and charlatans are lurking like spiders waiting for their prey." He dwells also on the pity of it that the poor should waste time and money on such practices while neglecting rational means of certain relief from duly qualified medical men.—*Journal A. M. A.,* Dec. 8, 1906.

